# Supplementary material for: An economic, square-shaped flat-field illumination module for TIRF-based super-resolution microscopy
Source: Biophys Rep (N Y). 2022 Jan 25;2(1):100044. doi: 10.1016/j.bpr.2022.100044 (PMC8914601; doi:10.1016/j.bpr.2022.100044)
Supplement: Document S1. Figs. S1–S3 and Tables S1 and S2 [file mmc1.pdf]

Biophysical Reports, Volume *2*

Supplemental information

An economic, square-shaped flat-field illumination module for TIRF-based super-resolution microscopy

Jeff Y.L. Lam, Yunzhao Wu, Eleni Dimou, Ziwei Zhang, Matthew R. Cheetham, Markus Körbel, Zengjie Xia, David Klenerman, and John S.H. Danial

Supplementary Information

An economic, square-shaped flat-field illumination module for TIRF-based super-resolution microscopy

Jeff Y.L. Lam[1,2,*], Yunzhao Wu[1,2,*], Eleni Dimou[1,2], Ziwei Zhang[1], Matthew R. Cheetham[1,2], Markus Körbel[1], Zengjie Xia[1,2], David Klenerman[1,2], John S.H. Danial[1,2]

[1] Yusuf Hamied Department of Chemistry, University of Cambridge, Cambridge CB2 1EW, UK.
[2] UK Dementia Research Institute, University of Cambridge, Cambridge CB2 0AH, UK.

[*] These authors contributed equally to this work.

Correspondence to John S H Danial (js2494@cam.ac.uk) or David Klenerman (dk10012@cam.ac.uk).

Supplementary figures

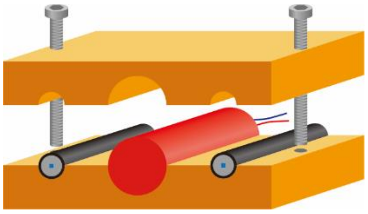

Figure S1. Schematic diagram of the vibrator in the home-built TIRF microscope with flat-field illumination.

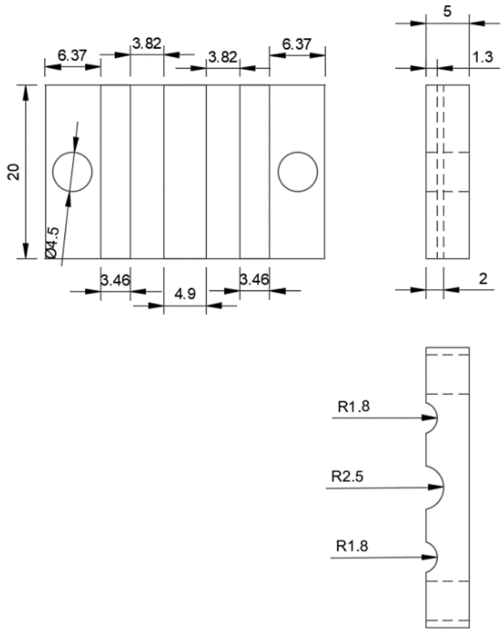

Figure S2. 2D sketch of the vibrator mount in the home-built TIRF microscope with flat-field illumination. All measurements are in millimetres. R – radius.

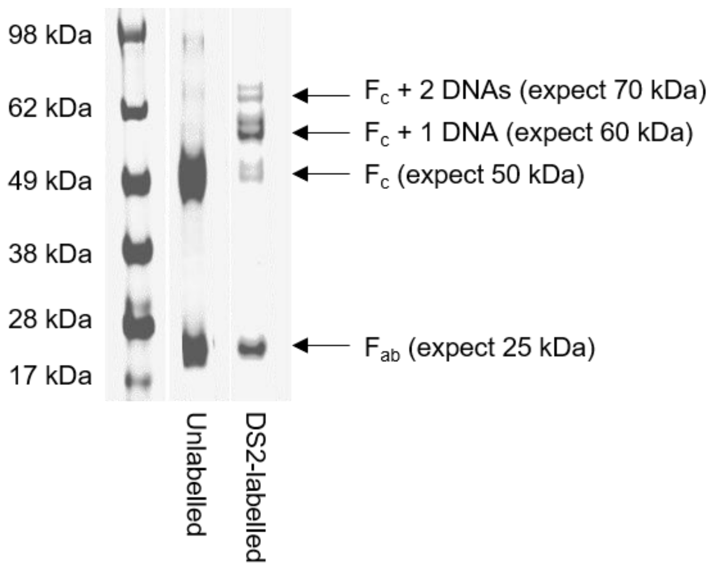

Figure S3. Characterization of DNA-labelled mouse anti-rabbit antibody by SDS-PAGE under reducing conditions.

Supplementary tables

Method	Wavelength	Ratio of power at focal plane to power at source
Free space launching	405 nm	7%
Free space launching	488 nm	6%
Free space launching	561 nm	12%
Free space launching	640 nm	9%
MMF	405 nm	55%
MMF	488 nm	55%
MMF	561 nm	56%
MMF	640 nm	46%

Table S1. Comparison between transmission efficiencies in a free space launching system (with the beam sufficiently expanded to simulate a flat-field) and the square-core MMF.

Code	Sequence (5’ - 3’)	Application
DBCO-DS2	DBCO TEG-TTATCTACATATTTTTTTTTTTTTTTTTTTT	Labelling of mouse anti-rabbit antibody
IS2-cy3B	TATGTAGATC-cy3B	Cellular DNA-PAINT
Aptamer-DS1	GCCTGTGGTGTTGGGGCGGGTGCGTTATACATCTA	AD-PAINT
IS1-cy3B	CTAGATGTAT-cy3B	AD-PAINT

Table S2. List of oligonucleotides and their applications. Underlined nucleotides are the complementary sequences that bind transiently to the docking strand and give a 9 bp DNA duplex during DNA-PAINT imaging. DBCO TEG – dibenzocyclooctyne tetraethylene glycol
