# Supplementary material for: An economic, square-shaped flat-field illumination module for TIRF-based super-resolution microscopy
Source: Biophys Rep (N Y). 2022 Jan 25;2(1):100044. doi: 10.1016/j.bpr.2022.100044 (PMC8914601; doi:10.1016/j.bpr.2022.100044)
Supplement: Document S2. Article plus supporting material [file mmc3.pdf]

Biophysical Reports

ARTICLE

An economic, square-shaped flat-field illumination module for TIRF-based super-resolution microscopy

Jeff Y. L. Lam,[1,2,3] Yunzhao Wu,[1,2,3] Eleni Dimou,[1,2] Ziwei Zhang,[1] Matthew R. Cheetham,[1,2] Markus Körbel,[1] Zengjie Xia,[1,2] David Klenerman,[1,2,*] and John S. H. Danial[1,2,*]
[1]Yusuf Hamied Department of Chemistry, University of Cambridge, Cambridge CB2 1EW, United Kingdom and [2]UK Dementia Research Institute, University of Cambridge, Cambridge CB2 0AH, United Kingdom

ABSTRACT Super-resolution microscopy allows complex biological assemblies to be observed with remarkable resolution. However, the presence of uneven Gaussian-shaped illumination hinders its use in quantitative imaging or high-throughput assays. Methods developed to circumvent this problem are often expensive, hard to implement, or not applicable to total internal reflection fluorescence imaging. We herein demonstrate a cost-effective method to overcome these challenges using a small square-core multimodal optical fiber as the coupler. We characterize our method with synthetic, recombinant, and cellular systems imaged under total internal reflection fluorescence and highly inclined and laminated optical sheet illuminations to demonstrate its ability to produce highly uniform images under all conditions.

WHY IT MATTERS Total internal reflection fluorescence (TIRF) microscopy is indispensable to biological imaging. However, it relies on the use of expensive fiber-based laser engines that deliver low laser powers (less than 50 mW) at non-uniform distributions and on a cost of 30,000 to 50,000 euros. In this work, we describe a novel fiber-based system that can deliver uniform, square-shaped illumination for high-quality TIRF microscopy. Importantly, our system can be used with cheap, high-power laser diodes to build a high-quality laser engine that costs 3,000 euros (i.e., 10 to 15 times cheaper than existing commercial solutions). Given the cost, quality, and ease of assembly, we expect our system to be quickly adopted on TIRF microscopes worldwide.

INTRODUCTION

Super-resolution (SR) microscopy has led to fundamental breakthroughs in biology by allowing cellular structures smaller than the diffraction limit of light to be readily observed (1–3). Single-molecule localization microscopy (SMLM) techniques, such as (direct) stochastic optical reconstruction microscopy (4), photoactivated localization microscopy (5), and DNA points accumulation for imaging in nanoscale topography (DNA-PAINT) (6), achieve SR by switching the emission of individual fluorophores one at a time and recording the position of each fluorophore down to a single-digit nanometre precision through the fitting of a point spread function (PSF). By virtue of its high resolving power and versatility, SMLM has been a highly popular tool employed worldwide.

To fully utilize the camera chip for imaging large samples by SMLM techniques, a full field of view (FOV) needs to be illuminated. As such, illumination homogeneity crucially affects the quality of a super-resolved image (7–9). In (direct) stochastic optical reconstruction microscopy and DNA-PAINT, the on-rate of fluorophores depends heavily on the intensity of the excitation source (7,9). Hence, uneven illumination results in a non-uniform distribution of emitted photons across the FOV and unwanted imaging artifacts. Poorly illuminated regions give rise to unclear structures, while strongly illuminated regions result in imprecise localizations that hamper quantitative analysis and molecular counting (7,10). In conventional SMLM setups, the excitation source is coupled to the objective by free-space optics, leading to a non-homogenous Gaussian-shaped illumination profile that induces the above problems. Recent attempts were made to reshape Gaussian-shaped beams into

Submitted November 18, 2021, and accepted for publication January 21, 2022.

[3]These authors contributed equally

*Correspondence: dk10012@cam.ac.uk or js2494@cam.ac.uk

Editor: Jörg Enderlein.

https://doi.org/10.1016/j.bpr.2022.100044

© 2022 The Author(s).

This is an open access article under the CC BY license (http://creativecommons.org/licenses/by/4.0/).

uniform flat-field illumination sources using microlens arrays (7), diffractive beam shaping devices (10), refractive beam shapers (9), and optical waveguides (11). Unfortunately, the application of these methods is hindered by either high costs or complicated optical setups.

Multi-mode fibers (MMFs) provide alternative means to obtaining flat-field illumination. As MMFs allow the transmission of multiple spatial modes, they produce even illumination at a high coupling efficiency (8). MMFs circumvent the drawbacks of using free-space-based flat-field illumination including the high cost and the requirement for the fine alignment of laser lines at the sample plane. It was earlier reported that a small circular core (50 and 105 μm) MMF could deliver a flat-field illumination suitable for total internal reflection fluorescence (TIRF) imaging but at the expense of producing a non-square FOV as well as a non-homogenous beam profile due to poor mode scrambling (12). More recently, a large square-core (150 × 150 μm) MMF was used to deliver a square flat-field illumination pattern for EPI-based experiments but not applicable under TIRF imaging due to the large size of the fiber-core diameter (13). Imaging under TIRF can potentially give a better signal-to-noise ratio, as only the fluorophores bound on the surface within the evanescent field will selectively be excited. This is especially important when there is an elevated background fluorescence, such as in DNA-PAINT imaging. Yet, none of the developed solutions with MMFs can deliver a square flat-field illumination that is suitable for surface-based SR imaging.

In this study, we demonstrate an economic flat-field illumination for TIRF-based SR microscopy by utilizing a small square-core (70 × 70 μm) MMF in the combined excitation path. There are two advantages to using a square-core fiber:

1. It enables the full utilization of square and rectangular camera chips.
2. It delivers improved mode scrambling compared with circular-core fibers (13). This is fundamentally important for minimizing the localization precision and improving stoichiometry quantification across the FOV as well as for the faithful representation of the underlying biological samples.

We systematically characterize the performance of our MMF with various samples, including fluorescent microspheres, CellMask Orange Plasma membrane stained T cells, supported lipid bilayers, synthetic DNA origamis, cellular microtubules, and recombinant protein aggregates illuminated under highly inclined and laminated optical (HILO) and TIRF. Our versatile and cost-efficient system can be easily assembled, aligned, and maintained to provide a square-shaped flat-field illumination.

MATERIALS AND METHODS

Home-built TIRF microscope with flat-field illumination setup

Four lasers operating at 405 (LDM-405-350-C, Lasertack GmbH), 488 (Toptica iBeam smart, Toptica), 561 (Cobolt Jive, HÜBNER) and 638 nm (Cobolt 06-MLD-638, HÜBNER) were coupled to the optical axis of a 1.49 N.A. 100x CFI Apo TIRF objective (MRD01991, Nikon) mounted on an inverted Ti-E Eclipse microscope (Nikon, Japan). The laser powers were controlled by their corresponding software or attenuated by neutral density filters. The laser beams then passed through the aligning mirrors and were combined by their corresponding dichroic mirror (for 405 nm: FF458-Di02-25x36, Semrock; for 488 nm: FF552-Di02-25x36, Semrock; for 561 nm: FF605-Di02-25x36, Semrock) before being focused by an aspheric lens (C220TMD-A, Thorlabs) to the square-core optical fiber (05806-1 Rev. A, CeramOptec). Launching was optimized using a free space fiber launch system (KT120/M, Thorlabs). Speckles from the fiber were removed using a vibration motor (304-111, Precision Microdrives) mounted on a custom 3D-printed mount (Data S1; Figs. S1 and S2). The mount was fixed to the fiber using two M4 screws and nuts 10 cm away from the beam collimator (see below). The combined laser beam coming out from the optical fiber was then collimated (C40FC-A, Thorlabs) and cleaned up by a quad-band excitation filter (FF01-390/482/563/640-25x36). To mechanically decouple the collimator from the microscope body, one thick (OR26X2V175, Hooper) or two thin (OR26X1V175, Hooper) Viton O rings were inserted between the external thread of the collimator and the internal thread of the mating optomechanic. Mechanical vibrations were ensured to be eliminated this way. The cleaned, collimated beam was then passed through the back port of the microscope and landed on an achromatic doublet lens (AC254-125-A-ML, Thorlabs). The excitation beam was then reflected by a quad-band dichroic beam splitter (Di01-R405/488/561/635-25x36, Semrock) or a penta-band dichroic beam splitter (R405/488/561/635/800-T1-25x36, Semrock) and focused on the sample by the objective. The size of the excitation beam can be adjusted by adjusting the distance between the collimator and back focal plane. Alternatively, different collimators (e.g., C80SMA-A, Thorlabs) can be used to control the size of the beam. Furthermore, due to geometrical constraints on the described setup, the collimated fiber output could have not been placed in a conjugate image plane. This results in the square profile being blurred at the sample with the central region exhibiting a flat, uniform intensity. On a custom microscope body, placing the beam in a conjugate image plane would result in a sharper square-shaped flat-field profile. Fluorescence from the sample was collected by the objective and passed through a quad-band emission filter (FF01-446/523/600/677-25x36, Semrock) and their corresponding appropriate filters (for both 405- and 488-nm-induced fluorescence: BLP01-488R-25x36, Semrock and FF01-520/44-25x36, Semrock; for 561-nm-induced fluorescence: LP02-568RS-25x36, Semrock and FF01-587/35-25x36, Semrock; for 638-nm-induced fluorescence BLP01-635R-25x36, Semrock) mounted on a high-speed filter wheel (HF110A, Prior Scientific) before being recorded on an emCCD camera (Evolve 512, Photometrics) operating in frame transfer mode (electron-multiplying gain of 3.1 electrons/analog-to-digital units [ADU] and 250 ADU/photon). Each pixel corresponds to a length of 105.4, 106.3, or 101.2 nm on the recorded image. The microscope was also fitted with a perfect focus system that auto-corrects the *z*-stage drift during a prolonged imaging period. To remove the stray infrared laser

beam, a short-pass filter (FESH0750, Thorlabs) was mounted on the entrance port of the emCCD camera.

Home-built TIRF microscope with Gaussian illumination setup

Four lasers operating at 405 (Cobolt 06-MLD-405, HÜBNER), 488 (Toptica iBeam smart, Toptica), 561 (Cobolt 06-DPL-638, HÜBNER), and 638 nm (Cobolt 06-MLD-638, HÜBNER) were coupled to the optical axis of a 1.49 N.A. 100x CFI Apo TIRF objective (MRD01991, Nikon) mounted on an inverted Ti-E Eclipse microscope (Nikon, Japan). The laser power was controlled by their corresponding software or attenuated by neutral density filters. The laser beams then passed through a quarter-wave plate for circular polarization and were cleaned up by their corresponding excitation filter (for 405 nm: FF01-417/60-25x36, Semrock; for 488 nm: LL01-488-25x36, Semrock; for 561 nm: FF01-561/14-25x36, Semrock; for 638 nm: FF01-640/14-25x36, Semrock). The laser beams were then expanded and collimated by beam expanders before reaching their corresponding dichroic mirror (for 405 nm: FF458-Di02-25x36, Semrock; for 488 nm: FF552-Di02-25x36, Semrock; for 561 nm: FF605-Di02-25x36, Semrock). Next, the reflected and combined laser beams were focused by an achromatic doublet lens (AC254-400-A, Thorlabs) and reflected by a quad-band dichroic beam splitter (Di01-R405/488/561/635-25x36, Semrock). The objective then focused the reflected excitation beam on the sample. Fluorescence was collected by the objective and passed through the quad-band dichroic beam splitter. It was then cleaned up by corresponding appropriate emission filter (for both 405- and 488-nm-induced fluorescence: BLP01-488R-25x36, Semrock and FF01-520/44-25x36, Semrock; for 561-nm-induced fluorescence: LP02-568RS-25x36, Semrock and FF01-587/35-25x36, Semrock; for 638-nm-induced fluorescence BLP01-635R-25x36, Semrock) mounted on a high-speed filter wheel (HF110A, Prior Scientific) before being recorded on an emCCD camera (Evolve 512, Photometrics) operating in frame-transfer mode (electron-multiplying gain of 3.1 electrons/ADU and 250 ADU/photon). Each pixel corresponds to a length of 101.6 nm on the recorded image. The microscope was also fitted with a perfect focus system that auto-corrects the *z*-stage drift during a prolonged period of imaging. To remove the stray infrared laser beam, a short-pass filter (FESH0750, Thorlabs) was mounted on the entrance port of the emCCD camera. The transmission efficiencies between this setup and the one with the square-shaped MMF for flat-field illumination were measured using a handheld power meter (S121C and PM100D, Thorlabs) (Table S1).

Imaging conditions

Slides were fixed on a microscope stage and coupled to an objective using refractive index-matched low-autofluorescence immersion oil (refractive index $n = 1.518$; Olympus, UK). Images were taken in a grid using an automation script (μManager v.1.4.22). Exposure times were set at 50 ms for diffraction-limited images for α-synuclein fibrils with thioflavin-T, 200 ms for diffraction-limited images for CellMask Orange Plasma membrane stained T cells, 33 ms for dSTORM, 100 ms for DNA-PAINT for DNA origami nanoruler (40 nm, cy3B, 40Y), immobilized high-resolution DNA-PAINT nanoruler (GATTAquant, catalog [cat.] no. 3030), and 150 ms for DNA-PAINT of microtubules in cells and AD-PAINT. For DNA-PAINT imaging of the nanoruler, 11,000 frames were acquired; for cellular dSTORM and DNA-PAINT imaging, 45,000 and 60,000 frames were acquired, respectively; for AD-PAINT imaging, 10,000 frames were recorded; for diffraction-limited images for α-synuclein fibrils with thioflavin-T and CellMask Orange Plasma membrane stained T cells, 50 and 100 frames were acquired, respectively.

PSF characterization of home-built TIRF microscope with flat-field illumination setup with fluorescent microspheres

TetraSpeck microspheres, 0.1 μm, fluorescent blue/green/orange/dark red (ThermoFisher, T7279) were embedded in 2% agarose gel (Merck, cat. no. A9414) on a glass coverslip in a ratio of 1:500. The sample was then excited by a 488 nm laser. The raw images were sliced, and full widths at half maximum (FWHMs) were calculated. The final calculated FWHMs were obtained by averaging 10 beads of different FOVs.

Imaging of beam profile

Three 15 cm extension tubes (SM1E60, Thorlabs) were connected and installed in front of the collimator in our home-built TIRF microscope with flat-field illumination setup. The beam profiles from different laser lines were recorded with a CMOS camera (DCC1545M-GL, Thorlabs) controlled with ThorCam software.

Preparation of lipid vesicle suspension

To 40 μL 25 mg/mL 1-palmitoyl-2-oleoyl-*sn*-glycero-3-phosphocholine (Avanti, cat. no. 850457C-200mg) solution in chloroform, 14 μL 1 mg/mL Oregon Green 488 1,2-dihexadecanoyl-*sn*-glycero-3-phosphoethanolamine (Invitrogen, Cat. No. 10154432) solution in chloroform was introduced. The mixture was then dried with a gentle flow of nitrogen to give a thin layer of lipids on the inner surface of a glass vial (Supelco, cat. no. 27134). The vial was protected from light and kept under vacuum overnight at room temperature to remove the residual chloroform. Next, 1 mL 18.2 MΩ cm water was added to the vial. The mixture was then vortexed for 1 min with a vortex mixer (PV-1, Grant Bio), after which the lipids were suspended in the water, forming multi-lamellar vesicles ("onions") presenting as a green-tinted cloudy suspension. The lipid suspension was sonicated for 20 min in an ice water bath with a 2 mm titanium probe (Sonicator Microprobe 4423, Qsonica) mounted on a tip sonicator (Ultrasonic Processor Q125, QSonica) with 60% maximum power and 45/15 s on/off cycle until it yielded a light green clear suspension, indicating the formation of small unilamellar lipid vesicles (SUVs) ∼20–25 nm in size. The SUV suspension was centrifuged at 14,100 × *g* for 2 min (Centrifuge 5418 R, Eppendorf) to remove titanium particles, and the supernatant was retained and stored at 4°C until use.

Surface treatment of glass coverslips for the formation of supported lipid bilayers

Glass coverslips (VWR, cat. no. MENZBC026076AC40) were cleaned by sonication (Ultrasonic cleaner USC100T, VWR) for 5 min in 18.2 MΩ cm water, 5 min in 2-propanol (Sigma-Aldrich, cat. no. 34863-2.5L), and 5 min in 18.2 MΩ cm water. The coverslips were then treated for 8 min with piranha solution, which was prepared by mixing 12 mL 98% sulfuric acid (Merck, cat. no. 5438270100) and 4 mL 30% hydrogen peroxide solution (Sigma-Aldrich, cat. no. 95321-100ML). Next, the piranha-treated coverslips were rinsed with excess 18.2 MΩ cm water and dried with a stream of nitrogen. A 50-well PDMS gasket (Merck, cat. no. GBL103250) was brought into conformal contact with the coverslip. Another 50-well PDMS gasket was then aligned and brought into conformal contact with the top of the first gasket. The chambered coverslip was kept in a humidity chamber and used within 10 min. Specifically, the humidity chamber was made by adding 10 mL 18.2 MΩ cm water to the bottom of an empty 250 μL pipette tip box (Rainin, cat. no. 30389193).

Formation of supported lipid bilayers

The SUV suspension in water was mixed with phosphate-buffered saline (PBS; ThermoFisher, cat. no. 10010023) at a ratio of 1:1 directly before use. The diluted SUV suspension (15 μL) was immediately added to each well of a chambered coverslip and incubated at 25°C for 10 min in a humidity chamber. Next, 8 μL of the suspension was drawn from each well, followed by the addition of 8 μL 18.2 MΩ cm water. This rinsing step was repeated three times. Then, 8 μL of the suspension was drawn from each well, after which 8 μL PBS was added. This step was also repeated three times before the coverslip was ready for imaging.

J8 LFA-1 Jurkat T cell sample preparation

The J8 LFA-1 Jurkat T cell line used was kindly provided by the Davis lab at the University of Oxford. The cells were grown at 37°C in 5% CO_2 in RPMI 1640 Medium, GlutaMAX Supplement, supplemented with 10% fetal calf serum, 10 mM HEPES, 1 mM sodium pyruvate, and 1% penicillin-streptomycin. The cell suspension was centrifuged at 2,000 Rpm for 2 min. The supernatant was removed, and the cell pellet was resuspended in 20 μL 1× PBS. The cells were then stained with CellMask Orange Plasma membrane (ThermoFisher, cat. no. C10045, 1:10,000 in PBS) for 10 min at 37°C, followed by the addition of 980 μL 1× PBS. The suspension was resuspended in 1 mL 1× PBS. The centrifugation-resuspension step was repeated three times to remove the excess dye. Finally, the cells were harvested and resuspended in 50 μL 1× PBS, which was then added to the poly-L-lysine-coated coverslip for imaging.

Preparation of poly-L-lysine-coated glass coverslip for CellMask Orange Plasma membrane stained T cells imaging

A 24 × 50 mm rectangular glass coverslip (VWR, cat. no. 631-0146) was cleaned with argon plasma (PDC-002, Harrick Plasma) for 1 h. Two pieces of 8-well PDMS gasket (Merck, cat. no. GBL103280) were aligned and attached to the coverslip. The glass surface was then treated with 1 mg/mL poly-L-lysine solution (Merck, cat. no. P8920-100ML) for 5 min. Each well was washed with 1× PBS (0.02-μm-filtered) three times before the suspension of stained cells was added.

Additional imaging conditions of CellMask Orange Plasma membrane stained T cells

The CellMask Orange Plasma membrane stained T cells were imaged with different optical fibers installed on our home-built TIRF microscope with flat-field illumination setup. The images of the same FOV were acquired with three optical fibers (05806-1 Rev. A, CeramOptec; M42L01, Thorlabs; M122L02, Thorlabs) sequentially installed on the system. The sample was excited by the 561 nm laser. The acquired images were stacked and averaged.

Characterization of the evanescent field of the home-built TIRF microscope with flat-field illumination setup

To measure the penetration depth of the TIRF illumination field, a raisin-cake fluorescent microsphere sample was prepared as previously described (21). Briefly, TetraSpeck fluorescent microspheres (ThermoFisher, T7279) were diluted 1:4 in 18.2 MΩ cm water. A drop of the diluted microspheres was then deposited onto a glass coverslip (VWR, cat. no. 631-0146) and allowed to dry. The coverslip was rinsed gently with 18.2 MΩ cm water to remove the excess microspheres. Meanwhile, a solution containing 0.5% agarose (Merck, cat. no. A9414) and 842 mM sucrose (Sigma-Aldrich, cat. no. 84100-1KG) was prepared, heated to 80°C, and slowly cooled down to 50°C. Then, 5 μL of TetraSpeck microspheres were sonicated for 10 s (Ultrasonic Cleaner USC100T, VWR), mixed with 15 μL of the agarose-sucrose solution, and immediately deposited on the coverslip at the same position where the microspheres were dried. The coverslip was placed in a humidity chamber and kept at 4°C for 2 h, allowing the agarose to polymerise. Finally, approximately 500 μL of the agarose-sucrose solution, which was kept at 30°C, was added onto the top of the microsphere-containing drop and allowed to polymerize at 4°C overnight in a humidity chamber. This sample was imaged on our flat-field setup with the 488 nm laser. For the same FOV, images were acquired from 2.5 μm below the surface to 5 μm over the surface with a *z*-step of 25 nm under EPI and TIRF illumination, respectively. The intensity profile of the fluorescent microspheres was extracted with ImageJ/Fiji (17). Data analysis was performed as described (21).

Preparation of oligonucleotides

All oligonucleotides (Table S2) were purchased from ATDBio (Southampton, UK). They were synthesized on the 1.0 μmol scale and purified by high-performance liquid chromatography, except for cy3B-labeled imaging strands, i.e., IS1 and IS2, which were purified by double high-performance liquid chromatography. Lyophilized oligonucleotides were dissolved in 18.2 MΩ cm water (filtered by 0.02 μm filter [VWR, cat. no. 516-1501]) to concentrations of 50–1,000 μM as confirmed by A_{260}, aliquoted, and stored at −20°C.

Antibody labeling for DNA-PAINT imaging

DBCO-DS2 was selectively conjugated on the carbohydrates of the fragment crystallizable region of the mouse anti-rabbit secondary antibody (Invitrogen, cat. no. 31213, lot no. UE2766762). To achieve this, the antibody was first functionalized using a SiteClick Antibody Azido Modification Kit (Invitrogen, cat. no. S20026) according to the manufacturer's instructions. Briefly, 250 μg antibody was concentrated to 3.4 mg/mL, and the buffer was exchanged in the provided antibody preparation buffer. The antibody was then incubated overnight with β-galactosidase at 37°C, followed by overnight coupling to UDP-GalNAz using β-1,4-galactosyltransferase on the next day at 30°C. The mixture was then purified by an Amicon spin filter (50 kDa molecular weight cut-off). The concentration of the azido-modified antibody was calculated by A_{280} (1.9 mg/mL). With the azido-modified antibody, 10 molar equivalents of DBCO-DS2 were introduced for copper-free strain-promoted click reaction in 1× PBS. After overnight incubation at 37°C, the excess oligonucleotide was removed using an Amicon spin filter (100 kDa molecular weight cut-off), and the concentration of antibody and the degree of labeling (3.2 docking strands per antibody) were determined by A_{260}/A_{280}. The purity and labeling efficiency were further confirmed using SDS-PAGE under reducing conditions (Fig. S3).

HEK293T cell sample preparation

HEK293T cells were maintained in complete DMEM supplemented with 10% fetal calf serum, 100 U/mL penicillin, 100 μg/mL streptomycin and grown at 37°C, and 5% CO_2. The cells were plated in

μ-slide 8-well glass-bottom chambers (ibidi, cat. no. 80827) pretreated with 0.1% poly-L-lysine (Merck, cat. no. P8920) and allowed to adhere overnight in complete medium. The next day, each well was incubated for ~30 s with 200 μL extraction buffer containing 0.25% Triton and 0.1% glutaraldehyde in PEM buffer (80 mM PIPES, 5 mM EGTA, 2 mM $MgCl_2$, pH 6.8) at 37°C, and the cells were subsequently fixed using 200 μL fixation buffer containing 0.25% Triton and 0.5% glutaraldehyde in PEM buffer for 10 min at 37°C. The samples were then washed three times with PBS (Merck, cat. no. 806544) and incubated with 0.1% $NaBH_4$ in PBS for 7 min at room temperature. This was followed by a 30 min blocking step using blocking buffer containing 0.5% fish gelatin for dSTORM samples, with an addition of 1 mg/mL salmon sperm DNA (ThermoFisher, cat. no. AM9680) for DNA-PAINT samples. The microtubules were immunostained overnight using a rabbit anti-tubulin antibody (Abcam, cat. no. ab18251, Lot No. GR3240348-1) diluted 1:300 in blocking buffer. The next day, the cells were rinsed three times with PBS and subsequently incubated with either an Alexa-Fluor-647-labeled goat anti-rabbit immunoglobulin G (H + L) (Invitrogen, cat. no. A32733, lot no. UH283999) at a concentration of 4 μg/mL in the blocking buffer for dSTORM or a DS2-labeled mouse anti-rabbit antibody at a concentration of 2 μg/mL for DNA-PAINT for 30 min at room temperature. Finally, samples were rinsed three times with 1× PBS before the introduction of the imaging solution.

Imaging solution for dSTORM

The imaging solution for dSTORM was prepared as reported. Briefly, the following stock solutions were prepared:

- 0.1 M Tris supplemented with 20 mM NaCl, pH 8, filtered by 0.02 μm filter (VWR, cat. no. 516-1501), stored at 4°C (2× imaging solution of dSTROM)
- 25% glucose, stored at 4°C (2.5× imaging solution of dSTORM)
- 1 M cysteamine (Merck, cat. no. 30070) in 0.36 M HCl, stored at 4°C for no more than 1 week (20× imaging solution of dSTORM)
- GOD buffer (24 mM PIPES, 4 mM $MgCl_2$, 2 mM EGTA) at pH 6.8 and filtered by 0.02 μm filter, stored at 4°C
- 20 mg/mL glucose oxidase from *Aspergillus niger* (Merck, cat. no. G2133) in GOD buffer, centrifuge filtered with 0.22 μm filter (Merck, cat. no. UFC30GV0S), flash-frozen in liquid nitrogen, stored in −80°C (40× imaging solution of dSTORM)
- 5 mg/mL catalase (Merck, cat. no. C40) in GOD buffer, centrifuge filtered with 0.22 μm filter (Merck, cat. no. UFC30GV0S), flash-frozen in liquid nitrogen, stored in −80°C (125× imaging solution of dSTORM)

The final working imaging solution for dSTORM contains 0.5 mg/mL glucose oxidase, 40 μg/mL catalase, 50 mM cysteamine, and 10% glucose in 50 mM Tris supplemented with 10 mM NaCl at pH 8. This solution was prepared freshly and immediately before imaging.

Preparation of recombinant α-synuclein aggregates

Wild-type α-synuclein was expressed, purified in *E. coli*, and stored at −80°C as described previously (14). To remove pre-aggregation seeds, the solution was centrifuged at 91,000 × *g* at 4°C for 1 h by an ultracentrifuge (Optima TLX Ultracentrifuge, Beckman). The concentration of the supernatant was then determined by A_{280} (ε_{280} = 5960 M^{-1} cm^{-1}). The supernatant was then diluted to 70 μM in 1× PBS supplemented with 0.01% NaN_3 (Merck, cat. no. 71290) and incubated at 37°C with shaking at 200 Rpm for 2 months.

Annealing of aptamer-DS1

The stock solution of aptamer-DS1 (1 mM) was diluted 10-fold into lithium cacodylate buffer, which contains 1 M KCl (Breckland, European Community no. 231-211-8, stock code: 0001276), 0.1 M cacodylic acid (Merck, cat. no. C0125), and 0.1 M lithium hydroxide (Merck, cat. no. 909025) in 0.02-μm-filtered 18.2 MΩ cm water, pH 7.3. The aptamer-DS1 solution at 100 μM was heated to 95°C for 10 min and then cooled down slowly overnight to room temperature.

AD-PAINT

AD-PAINT was performed as we described previously (15). Briefly, a 50 mm diameter round coverslip (VWR, cat. no. 631-0178) was cleaned with argon plasma (PDC-002, Harrick Plasma) for 1 h. A 50-well PDMS gasket (Merck, cat. no. GBL103250) was cut into halves and then affixed to the coverslip. The slide was then treated with 0.02-μm-filtered (VWR, cat. no. 516-1501) 1% Tween (Fisher Scientific, cat. no. BP337-100, lot no. 179118)/PBS (ThermoFisher, cat. no. 10010023) for 1 h. It was then rinsed three times with 1× PBS (0.02-μm-filtered). Two-month α-synuclein (280 nM) was then introduced onto the slide and incubated for 5 min. After incubation at room temperature, the sample was removed, and the wells were filled with the imaging solution. To avoid evaporation over prolonged imaging, another clean coverslip was layered on top of the PDMS gasket.

Imaging solution for DNA-PAINT

For cellular DNA-PAINT imaging

The imaging strand, IS2-cy3B, was in an oxygen-scavenging system PO + C. The oxygen-scavenging system was prepared as previously reported (16). Briefly, the following stock solutions were prepared.

- PO + C buffer (10 mM Tris pH 7.5, 50 mM KCl and 20% glycerol), stored at 4°C
- 38 mg/mL pyranose oxidase from *Coriolus sp.* (Merck, cat. no. P4234) in PO + C buffer (100× imaging solution for cellular DNA-PAINT imaging), centrifuge filtered with 0.22 μm filter (Merck, cat. no. UFC30GV0S), flash-frozen in liquid nitrogen, stored in −80°C
- 2 mg/mL catalase (Merck, cat. no. C40) in PO + C buffer, centrifuge filtered with 0.22 μm filter (Merck, cat. no. UFC30GV0S), flash-frozen in liquid nitrogen, stored in −80°C (100× imaging solution of cellular DNA-PAINT imaging)
- 100 mg Trolox (Merck, cat. no. 238813) was dissolved in 430 μL methanol, 345 μL NaOH (1 M), and 3.2 mL 0.02-μm-filtered (VWR, cat. no. 516-1501) 18.2 MΩ cm water, stored at −20°C (100× imaging solution of cellular DNA-PAINT imaging)

The final working imaging solution for cellular DNA-PAINT imaging contains 5 nM IS2-cy3B, 3.8 mg/mL pyranose oxidase, 20 μg/mL catalase, and 0.25 mg/mL Trolox.

For AD-PAINT imaging

The imaging solution was prepared as we previously reported (15). Briefly, ultrapure grade thioflavin-T (Anaspec, cat. no. AS-88306) was dissolved in 18.2 MΩ cm water at 100 μM as confirmed by A_{412} (ε_{412} = 31,600 M^{-1} cm^{-1}). The thioflavin-T solution was then filtered by a 0.02 μm filter (VWR, cat. no. 516-1501), stored at 4°C in dark for no more than a month. The final working imaging solution for AD-PAINT contains 1 nM IS1-cy3B, 100 nM Aptamer-DS1, and 5 μM thioflavin-T solution in PBS (ThermoFisher, cat. no. 10010023).

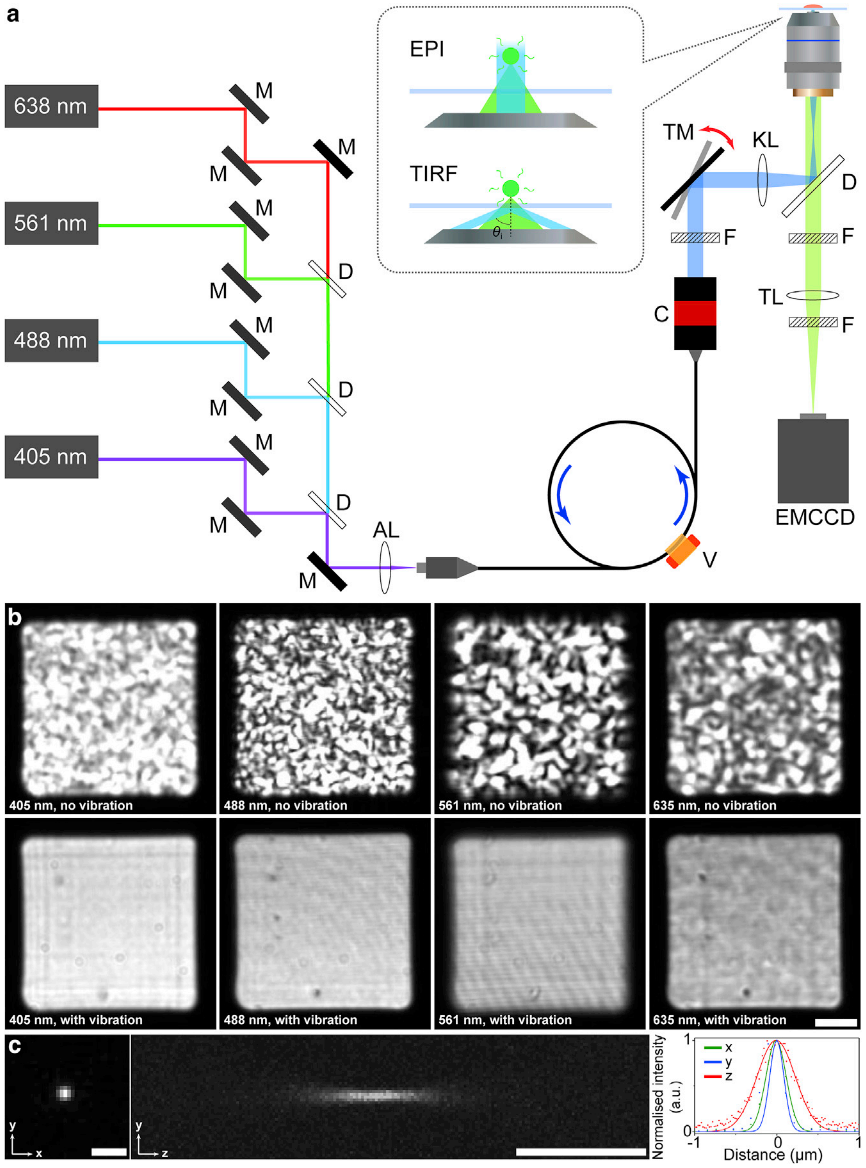

FIGURE 1 Schematic diagram of our home-built TIRF microscope with flat-field illumination and its demonstration as well as resolution characterization. (*a*) Schematic diagram of the setup. Solid black filled rectangles: M represents dielectric mirrors for aligning; C represents the fiber collimator; V represents the vibration motor; TM shows the dielectric mirror for adjusting the angle of incident beam θ; black unfilled rectangles: D represent dichroic mirrors; striped rectangles: F represent filters; ellipses AL, KL, and TL represent aspheric lens, achromatic doublet lens, and tube lens, respectively. The single arrow indicates the direction of the combined laser beam while the double arrow indicates the alternation of TM. (*b*) Demonstration of the illumination profile after coupling into the square-core optical fiber without (top) and with (bottom) fiber agitation (i.e., vibration) for speckle reduction as imaged on a CMOS camera after collimation for all four different lasers. Scale bar, 10 μm. (*c*) Point spread function of our home-built TIRF microscope with flat-field illumination, measured by 100 nm microspheres under 488 nm excitation. Representative cross sections of the point spread function of the fluorescent microspheres are shown in the *x*-*y* plane (left) and in the *y*-*z* plane (middle). Intensity plots along the three axes (right) show that the full widths at half maximum are 293.4, 272.1, and 544.2 nm along the *x*, *y*, and *z* axes, respectively. Scale bars, 1 μm.

SR image reconstruction and data analysis

The positions of the transient bindings between the imaging and docking strands or photo-switching of the fluorophores, "blinkings," were determined by using either of the following:

- The PeakFit plugin, a plugin of the GDSC Single Molecule Light Microscopy package (http://www.sussex.ac.uk/gdsc/intranet/microscopy/imagej/gdsc_plugins) for imageJ/Fiji (17) with a typical "signal strength" threshold of 40 and a precision threshold of 20 nm at a magnification as 8.
- The thunderSTORM plugin (18) for ImageJ/Fiji (17) with magnification as 10, B-spline order and magnitude as 3 and 2.0, respectively, and fitting radius and initial sigma as 3 and 1.6 pixels, respectively.

To automate SR reconstruction for multiple images with the PeakFit plugin in image J/Fiji, a script working on the python 3.7 platform (https://github.com/Eric-Kobayashi/SR_toolkit), was applied.

The resolution was determined by plotting a Fourier ring correlation curve with the FIRE plugin (19) for imageJ/Fiji (17) for the image super reconstructed by thunderSTORM and determining the spatial frequency at which the curve drops below 1/7 (20).

Theoretical calculations: Contribution of small, square-core MMF to TIRF imaging

For a beam focused at the back focal aperture to effectively contribute to an evanescent field, the size of the beam S_b has to be smaller than the size of the objective lens TIRF annulus S_a.

S_a is given by $f_B(NA - n_s)$ (12) where f_b is the back focal length of the objective lens given by F_L/M, where F_L is the focal length of the standard tube lens and M is the magnification of the objective lens. NA is the numerical aperture of the objective lens, and n_s is the refractive index of the sample (i.e., PBS buffer). For the imaging system used in this work, F_L is 200 mm, M is 100—hence f_B is 2—,NA is 1.49, and n_s is 1.335. Therefore, S_a is 310 μm.

S_b is given by $S_c F_k / F_{cl}$, where S_c is the size of the fiber core, F_k is the focal length of the Koehler lens, and F_{cl} is the focal length of the beam

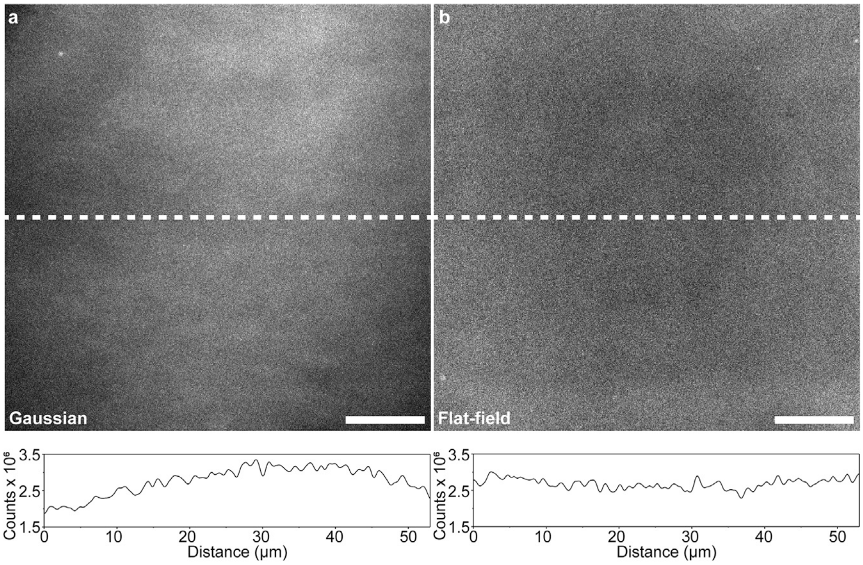

FIGURE 2 Characterizing flatness of the MMF-based illumination. (*a* and *b*) TIRF images of the full field of view (top) and line plots of fluorescence intensity along the principal axis (white, bottom) obtained by supported POPC lipid bilayer with 1% 1,2-dihexadecanoyl-*sn*-glycero-3-phosphoethanolamine under (*a*) conventional Gaussian and (*b*) flat-field illumination profiles. Although the Gaussianprofile represents the profile of a free-launched beam, the profile deviates from a perfect Gaussian shape as is common for some lasers. Scale bars, 10 μm.

collimator. For the imaging system used in this work, S_c is 70 μm, F_k is 125 mm, and F_{cl} is 40 mm. Therefore, S_b is 262.5 μm.

RESULTS

To achieve a square-shaped flat-field illumination profile, we introduced a small square-core (70 × 70 μm) MMF in the combined excitation path of our home-built TIRF microscope (Fig. 1 *a*; see Materials and methods). Briefly, the Gaussian-shaped output of four laser lines was coupled to the fiber using an aspheric lens at high coupling efficiencies (81% for 405 nm, 86% for 488 nm, 93% for 561 nm, and 82% for 638 nm). The output of the fiber was then collimated and focused onto the back focal plane of the objective using a combination of an adjustable-focus fiber collimator and an achromatic doublet lens to produce a square-shaped flat-field illumination profile at the sample plane (Fig. 1 *b*). Autofluorescence generated by the fiber was removed using a quad-band excitation filter, and speckles caused by interference were eliminated using a cylindrical vibration motor attached onto the MMF with a 3D-printed mount (Figs. 1 *a*, S1, and S2; see Materials and methods).

We first characterized the PSF of our flat-field illumination module by calculating the FWHM of the PSF obtained from 100 nm microspheres under 488 nm excitation. The measured lateral resolutions of our system were 294 ± 22 nm in the *x* direction and 286 ± 20 nm in the *y* direction, which are comparable to the resolutions of most conventional TIRF microscopes (21). The axial resolution of our system was 545 ± 15 nm (Fig. 1 *c*).

In our inverted fluorescence microscope system, the angle of the incident laser beam (θ in Fig. 1 *a*) can be varied easily with a manually tuneable mirror (TM in Fig. 1 *a*) at the back of the microscope. This allows us to switch between EPI illumination, HILO, and TIRF. With the application of a small-core optical fiber, the full laser beam can be totally reflected without any partial refraction that could otherwise cause unintended excitation of fluorophores in the background under TIRF-based imaging.

We then evaluated the homogeneity of the emission profile by imaging a supported lipid bilayer, a smooth surface consisting of two layers of lipid molecules that exhibits lateral fluidity, formed on a glass coverslip. To accomplish this, we doped 1% fluorescently labeled Oregon Green-1,2-dihexadecanoyl-*sn*-glycero-3-phosphoethanolamine into 1-palmitoyl-2-oleoyl-*sn*-glycero-3-phosphocholine. This gives rise to a uniformly fluorescent surface and therefore allows us to observe the emission profile of the microscope under Gaussian- and square-shaped flat-field illuminations (Fig. 2 *a* and *b*, respectively). Examining the acquired images, we uncovered a two-fold intensity difference between the center and peripheries of the FOVs under conventional Gaussian-shaped illumination (Fig. 2 *a*, *bottom*), while observing a highly uniform emission profile across the entire FOV under flat-field illumination (Fig. 2 *b*, *bottom*).

We first performed a comparative assessment of the suitability of our MMF for TIRF by imaging surface-adhered CellMask Orange Plasma membrane stained T cells using a 50 μm circular core MMF (solution proposed in (12); Fig. 3 *a* and *b*), 70 μm square core MMF (solution proposed here; Fig. 3 *c* and *d*), and 200 μm square core MMF (solution proposed in (13); Fig. 3 *e* and *f*). The 50 μm circular core and 70 μm square core MMFs show higher quality TIRF imaging compared

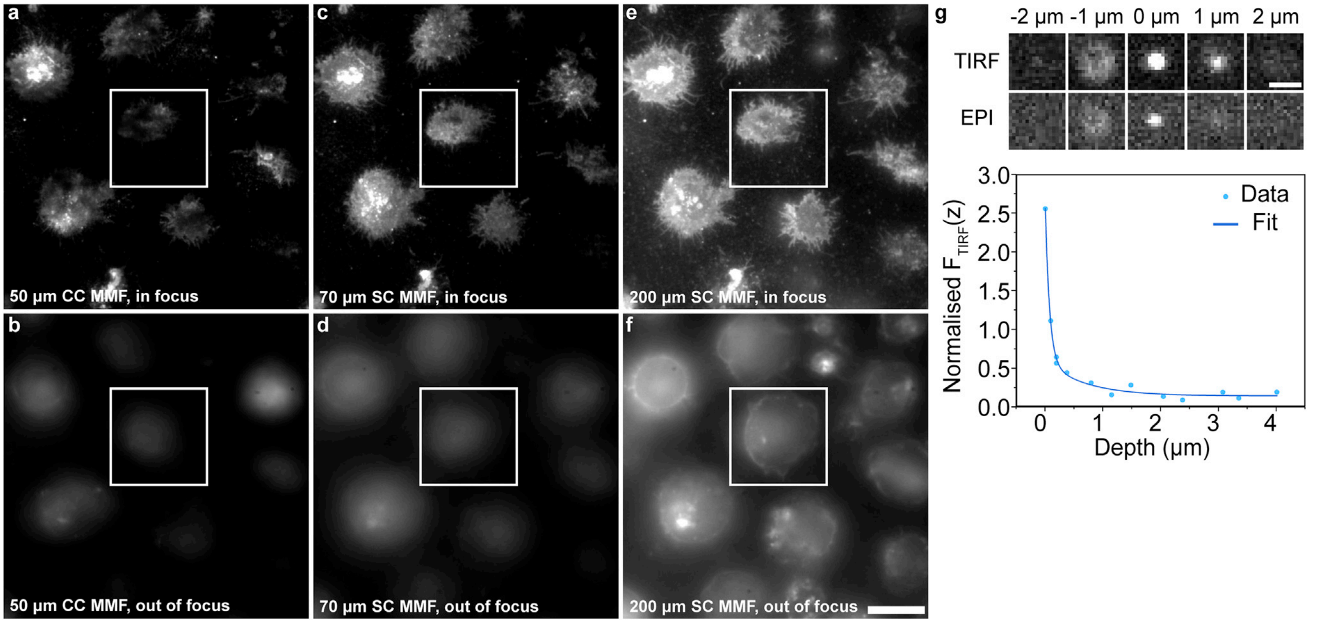

FIGURE 3 Qualitative and quantitative assessments of the MMF-based illumination for TIRF imaging. (*a–f*) Diffraction-limited images of surface-adhered CellMask Orange Plasma membrane stained T cells illuminated using a 561 nm laser line coupled into 50 μm circular core MMF (solution proposed in (12), *a* and *b*), 70 μm square core MMF (solution proposed here, *c* and *d*), and 200 μm square core MMF (solution proposed in (13), *e* and *f*). Images were acquired at the focal plane (*top*) and 4 μm above the focal plane (*bottom*). Images of fluorescent beads in a raisin cake at different depths under TIRF and EPI as well as a plot of the normalized TIRF intensity at different depths. Fitting the raw to a double exponential model (Eq. 3 in (22)) yields an angle-independent depth (D = 661.2 ± 188.9 nm) and an angle-dependent penetration depth (δ = 73.2 ± 13.1 nm). Scale bars, 10 μm (*a–f*) and 1 μm (*g*).

with the 200 μm square core MMF, which shows the appearance of membrane contours (Fig. 3 *f*) out of focus. Furthermore, the 50 μm circular core fiber shows an uneven illumination pattern (Fig. 3 *a*) compared with the 70 μm fiber (Fig. 3 *c*), which is presumably due to poor mode scrambling. Our theoretical calculations (see Materials and methods) show that our microscope objective has a 310 μm TIRF annulus and that the beam size at the back focal aperture is 262.5 μm. This confirms that the beam in its entirety contributes to the production of an evanescent field for TIRF imaging. Apart from this qualitative assessment of the suitability of our solution for TIRF imaging, we performed a quantitative assessment by imaging a raisin cake of fluorescent beads at different depths under EPI and TIRF to calculate the evanescent-field decay length (Fig. 3 *g*). Our measurements show an angle-dependent decay length of 73.2 nm, which is in excellent agreement with (and lower than) published values (22).

We then assessed the suitability of our system for SR imaging using DNA-PAINT. Successful DNA-PAINT imaging requires the sample of interest to be excited under TIRF to suppress the background of fluorescently labeled imaging strands and to illuminate the surface-bound species alone. To demonstrate the applicability of our fiber-based solution to TIRF-based DNA-PAINT imaging, we imaged surface-immobilized nanorulers (three-spot DNA origami nanostructures with 40 nm distance between each spot; Fig. 4 *a*) under EPI- and TIRF illumination (Fig. 5 *a* and *b*). A poor signal-to-noise ratio due to high background fluorescence was observed under EPI illumination (Fig. 5 *a*); on the other

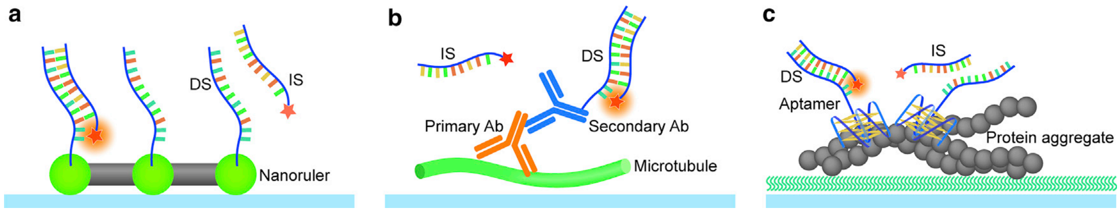

FIGURE 4 Schematic diagrams for DNA-PAINT. (*a–c*) DNA-PAINT on (*a*) origami nanostructures on nanorulers, (*b*) fixed cellular microtubules immunostained by DNA-antibody conjugates, and (*c*) aptamer (AD-PAINT)-targeting protein aggregates. DS, docking strand; IS, imaging strand; Ab, antibody.

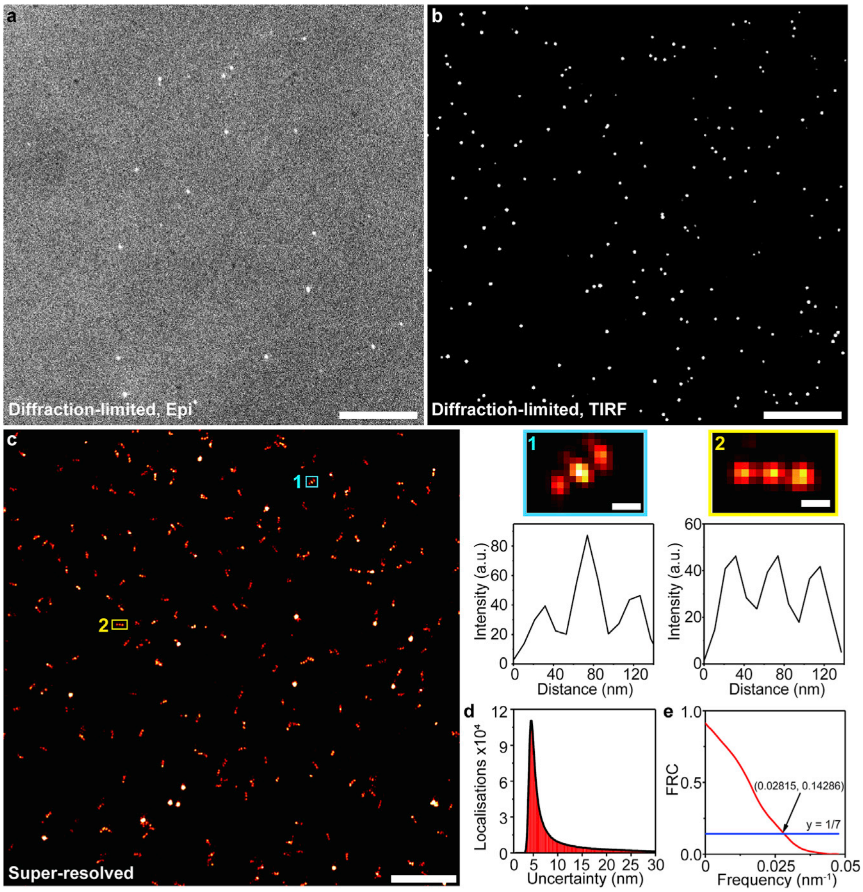

FIGURE 5 Diffraction-limited and super-resolved images of the nanorulers and their analyses. (*a* and *b*) Diffraction-limited images of nanorulers under (*a*) EPI- and (*b*) TIRF illumination. Both images were taken at the same plane with the same exposure settings and adjusted to the same level of brightness and contrast. A clear high background fluorescence (approximately six-fold) is observed (*a*) under EPI-illumination compared with (*b*) under TIRF illumination. Scale bars, 10 μm (*a* and *b*). (*c*) Super-resolved image of the three-spot DNA origami nanostructures with 40 nm distance between each spot with imaging strand attached to cy3B, with a laser power of 111 mW before the objective. The image was reconstructed and drift-corrected from 10,000 frames with an exposure time of 100 ms. Scale bar, 1 μm. (*1* and *2*) Close-up images of the corresponding regions in (*c*) (top, scale bar, 50 nm) and their corresponding intensity versus distance profile (*bottom*). (*d*) The number of localizations versus the localization uncertainty shows that we can achieve DNA-PAINT imaging with the nanorulers at a precision of 8.7 nm. (*e*) Fourier ring correlation measurement on DNA-PAINT images of nanorulers. This shows that the resolution obtained was 35.5 nm at which the curve drops below 1/7.

hand, an improved signal-to-noise ratio was obtained with TIRF, as the fluorescence intensity of the fluorophore bound on the surface was enhanced while that in the bulk outside the evanescent field was dramatically reduced (Fig. 5 *b*). This again demonstrates the ability of our fiber-based flat-field illumination module for TIRF-based imaging. Furthermore, we could resolve the nanorulers with fiducial markers across the entire FOV (Fig. 5 *c*) at a mean localization precision of 8.7 nm (Fig. 5 *d*). The image resolution was calculated to be 35.5 nm by Fourier ring correlation (Fig. 5 *e*). This demonstrates the ability of our setup to resolve nanoscopic structures with exceptional uniformity under TIRF illumination.

With the success of applying our fiber-based solution to imaging synthetic surface-based structures under TIRF illumination, we then used our system for visualizing cellular microtubules (Fig. 4 *b*) under HILO illumination to interrogate the entire cell volume. The microtubules were immunostained using antibodies either conjugated with dyes for dSTORM or docking strands for DNA-PAINT (see Materials and methods). Using our fiber-based, square-shaped flat-field module, a uniform localization density was maintained from the centers to the peripheries of the FOVs in both DNA-PAINT (Fig. 6 *a*) and dSTORM (Fig. 6 *b*) imaging under HILO. The images confirm the suitability of our solution for resolving nanoscopic cellular structures precisely under HILO illumination and uniformly across a wide FOV.

Having demonstrated the applicability of our fiber-based system to imaging synthetic and cellular samples, we applied it for imaging recombinantly prepared protein aggregates (Fig. 4 *c*) using aptamer DNA-PAINT (AD-PAINT). AD-PAINT is a recently developed technique for examining surface-captured protein complexes below the diffraction limit (16). Aptamers are single-stranded oligonucleotides that can fold into G-quadruplexes, a secondary nucleic acid structure formed among guanine bases by Hoogsteen-type hydrogen bonds. Some aptamers have a high affinity and specificity for different protein oligomers and fibrils, thus acting as tiny molecular probes (23). By introducing a DNA docking strand on an aptamer, which is complementary to a fluorescently labeled imaging strand, it is possible to perform DNA-PAINT on surface-captured protein aggregates. Here, we used an aptamer that recognizes fibrils of the intrinsically disordered protein α-synuclein, which is implicated in

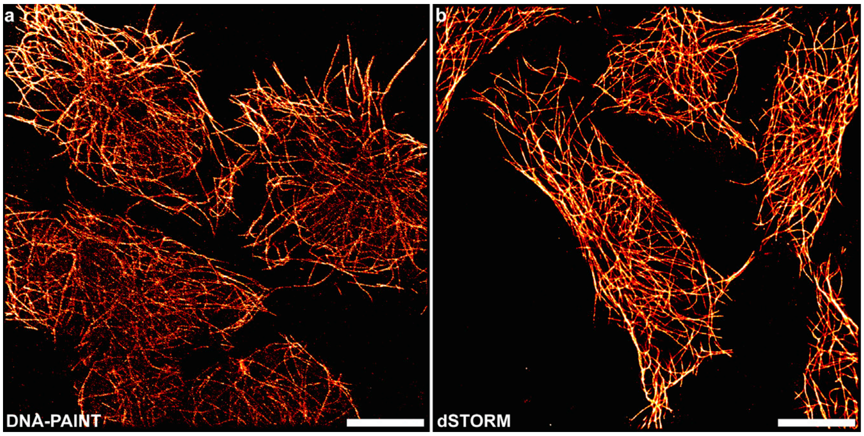

FIGURE 6 Super-resolution imaging of cellular samples. (*a* and *b*) Super-resolved, full field-of-view image of the microtubules in fixed and permeabilized HEK293T cells for (*a*) DNA-PAINT imaging and (*b*) dSTORM under highly inclined and laminated optical illumination in our fiber-based, square-shaped flat-field setup. Scale bar, 10 μm.

Parkinson's disease (16,23,24). We first imaged the fibrils at the diffraction limit using thioflavin-T, a dye that interacts with the hydrophobic regions of protein aggregates, to ensure their presence (Fig. 7 *a*) then super-resolved them using AD-PAINT (Fig. 7 *b*), all under TIRF illumination. As with the other samples, we observe uniform imaging of the fibrils across the entire FOV, demonstrating the suitability of our solution for high-throughput light-based assays.

DISCUSSION

Currently available options to generate a square flat-field illumination are dependent on the use of expensive beam shapers, which are not compatible with commercially available, fiber-based laser engines or large square-core fibers, which are only compatible with EPI/HILO illumination but not with TIRF. Our small square-core MMF is as follows:

1. Compatible with fiber-based laser engines (which are widely used in commercially available or home-built setups (25)).
2. Economic (1,000 compared with 5,000 USD for a beam shaper (10)). Although we have assessed the MMF with a combination of expensive, high-quality, low-power lasers and cheap, low-quality, high-power lasers, it is possible to use the proposed square-core MMF with cheap, high-power laser diodes of different wavelengths only to construct laser engines that are 10-fold cheaper than commercially existing alternatives.
3. Compatible with TIRF illumination (owing to the small size of its core compared with widely available square-core MMFs (13)).
4. Optimized for square and rectangular large camera chips (owing to the square core and its ability to illuminate large FOVs).
5. Compatible with highly homogenous illumination (owing to improved mode scrambling compared to circular-core MMFs (13)).

Our solution is, to our knowledge, novel and not only adds important value and innovation to the field but sets a standard for high-quality imaging at low cost and with very easy implementation. Due to these

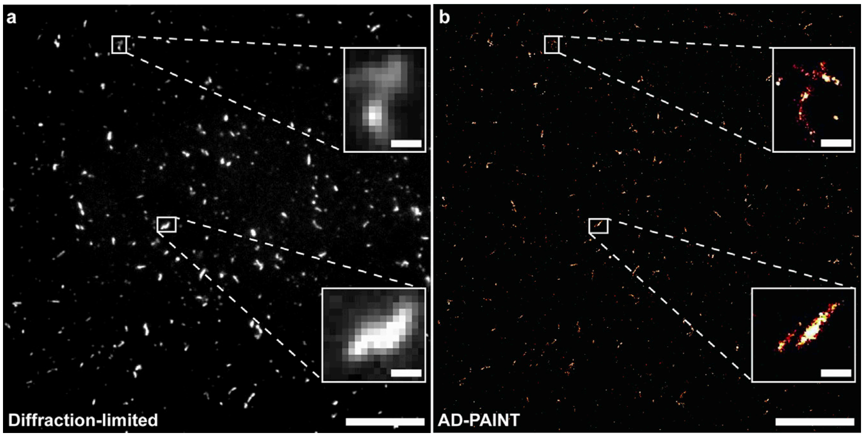

FIGURE 7 Super-resolution imaging of recombinant samples. (*a* and *b*) Diffraction-limited (*a*) and super-resolved (*b*) image of the α-synuclein fibrils with thioflavin-T and aptamer-docking strand DNA, respectively. The scale bars for (*a*) and (*b*) are 10 μm while that of the close-up in the (*a*) diffraction limited images and (*b*) super-resolved images are 500 nm.

attributes, we expect its quick adoption across academia and industry.

SUPPORTING MATERIAL

Supporting material can be found online at https://doi.org/10.1016/j.bpr.2022.100044.

AUTHOR CONTRIBUTIONS

J.S.H.D. conceived and designed the study. J.Y.L.L., Y.W., and Z.X. constructed the microscopy setups. J.Y.L.L., Y.W., E.D., Z.Z., M.R.C., and M.K. prepared all samples. J.Y.L.L. and Y.W. performed all analyses. J.Y.L.L. wrote the manuscript with input from all authors. D.K. supervised the project.

DECLARATION OF INTERESTS

The authors declare no competing interests.

ACKNOWLEDGMENTS

We would like to thank Jonas Ries (EMBL) for useful preliminary discussions. We would also like to thank Simon J. Davis and Edward Jenkins (University of Oxford) for providing the J8 LFA-1 Jurkat T cell line. J.Y.L.L. is supported by the Croucher Foundation Limited (Hong Kong). E.D. is funded by a Deutsche Forschungsgemeinschaft Research Fellowship (426806622). M.K. is funded by the Royal Society (RP/EA/180002). J.S.H.D is funded by a postdoctoral fellowship from Eisai and the UK Dementia Research Institute (DRI) Limited, a pilot grant from the UK DRI, and a research associateship from King's College, University of Cambridge. D.K. is funded by a European Research Council advanced grant (669237), the UK DRI, Ltd., funded by the UK Medical Research Council, and by the Royal Society (UK).

REFERENCES

1. Huang, B., M. Bates, and X. Zhuang. 2009. Super resolution fluorescence microscopy. *Annu. Rev. Biochem.* 78:993–1016.
2. Yue, L., H. Shen, …, L. Zhang. 2016. Image super-resolution: the techniques, applications, and future. *Signal Process.* 128:389–408.
3. Schermelleh, L., A. Ferrand, …, G. P. Drummen. 2019. Super-resolution microscopy demystified. *Nat. Cell Biol.* 21:72–84.
4. Rust, M. J., M. Bates, and X. Zhuang. 2006. Sub-diffraction-limit imaging by stochastic optical reconstruction microscopy (STORM). *Nat. Methods.* 3:793–796.
5. Betzig, E., G. H. Patterson, …, H. F. Hess. 2006. Imaging intracellular fluorescent proteins at nanometer resolution. *Science.* 313:1642–1645.
6. Schnitzbauer, J., M. T. Strauss, …, R. Jungmann. 2017. Super-resolution microscopy with DNA-PAINT. *Nat. Protoc.* 12:1198–1228.
7. Douglass, K. M., C. Sieben, …, S. Manley. 2016. Super-resolution imaging of multiple cells by optimized flat-field EPI-illumination. *Nat. Photonics.* 10:705–708.
8. Deschamps, J., A. Rowald, and J. Ries. 2016. Efficient homogeneous illumination and optical sectioning for quantitative single-molecule localization microscopy. *Opt. Express.* 24:28080–28090.
9. Rowlands, C. J., F. Ströhl, …, C. F. Kaminski. 2018. Flat-field super-resolution localization microscopy with a low-cost refractive beam-shaping element. *Sci. Rep.* 8:5630.
10. Stehr, F., J. Stein, …, R. Jungmann. 2019. Flat-top TIRF illumination boosts DNA-PAINT imaging and quantification. *Nat. Commun.* 10:1268.
11. Archetti, A., E. Glushkov, …, S. Manley. 2019. Waveguide-PAINT offers an open platform for large field-of-view super-resolution imaging. *Nat. Commun.* 10:1267.
12. Kwakwa, K., A. Savell, …, P. M. French. 2016. easySTORM: a robust, lower-cost approach to localisation and TIRF microscopy. *J. Biophotonics.* 9:948–957.
13. Schröder, D., J. Deschamps, …, J. Ries. 2020. Cost-efficient open source laser engine for microscopy. *Biomed. Opt. Express.* 11:609–623.
14. Jimenez, A., K. Friedl, and C. Leterrier. 2020. About samples, giving examples: optimized single molecule localization microscopy. *Methods.* 174:100–114.
15. Cremades, N., S. I. Cohen, …, D. Klenerman. 2012. Direct observation of the interconversion of normal and toxic forms of α-synuclein. *Cell.* 149:1048–1059.
16. Whiten, D. R., Y. Zuo, …, M. H. Horrocks. 2018. Nanoscopic characterisation of individual endogenous protein aggregates in human neuronal cells. *Chembiochem.* 19:2033–2038.
17. Blumhardt, P., J. Stein, …, P. Schwille. 2018. Photo-induced depletion of binding sites in DNA-PAINT microscopy. *Molecules.* 23:E3165.
18. Schindelin, J., I. Arganda-Carreras, …, A. Cardona. 2012. Fiji: an open-source platform for biological-image analysis. *Nat. Methods.* 9:676–682.
19. Ovesný, M., P. Křížek, …, G. M. Hagen. 2014. ThunderSTORM: a comprehensive ImageJ plug-in for PALM and STORM data analysis and super-resolution imaging. *Bioinformatics.* 30:2389–2390.
20. Nieuwenhuizen, R. P. J., K. A. Lidke, …, B. Rieger. 2013. Measuring image resolution in optical nanoscopy. *Nat. Methods.* 10:557–562.
21. Beck, M., M. Aschwanden, and A. Stemmer. 2008. Sub-100-nanometre resolution in total internal reflection fluorescence microscopy. *J. Microsc.* 232:99–105.
22. Oheim, M., A. Salomon, …, U. Becherer. 2019. Calibrating evanescent-wave penetration depths for biological TIRF microscopy. *Biophys. J.* 117:795–809.
23. Tsukakoshi, K., K. Abe, …, K. Ikebukuro. 2012. Selection of DNA aptamers that recognize α-synuclein oligomers using a competitive screening method. *Anal. Chem.* 84:5542–5547.
24. Spillantini, M. G., M. L. Schmidt, …, M. Goedert. 1997. α-Synuclein in Lewy bodies. *Nature.* 388:839–840.
25. Martens, K. J. A., S. P. van Beljouw, …, J. Hohlbein. 2019. Visualisation of dCas9 target search in vivo using an open-microscopy framework. *Nat. Commun.* 10:3552.

Biophysical Reports, Volume *2*

Supplemental information

An economic, square-shaped flat-field illumination module for TIRF-based super-resolution microscopy

Jeff Y.L. Lam, Yunzhao Wu, Eleni Dimou, Ziwei Zhang, Matthew R. Cheetham, Markus Körbel, Zengjie Xia, David Klenerman, and John S.H. Danial

Supplementary Information

An economic, square-shaped flat-field illumination module for TIRF-based super-resolution microscopy

Jeff Y.L. Lam[1,2,*], Yunzhao Wu[1,2,*], Eleni Dimou[1,2], Ziwei Zhang[1], Matthew R. Cheetham[1,2], Markus Körbel[1], Zengjie Xia[1,2], David Klenerman[1,2], John S.H. Danial[1,2]

[1] Yusuf Hamied Department of Chemistry, University of Cambridge, Cambridge CB2 1EW, UK.
[2] UK Dementia Research Institute, University of Cambridge, Cambridge CB2 0AH, UK.

[*] These authors contributed equally to this work.

Correspondence to John S H Danial (js2494@cam.ac.uk) or David Klenerman (dk10012@cam.ac.uk).

Supplementary figures

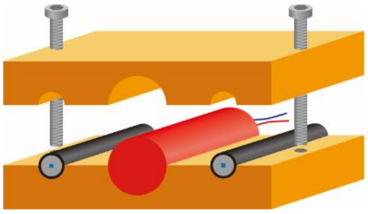

Figure S1. Schematic diagram of the vibrator in the home-built TIRF microscope with flat-field illumination.

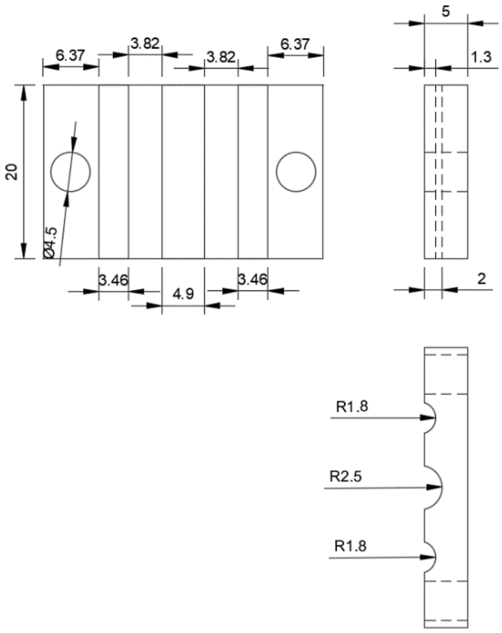

Figure S2. 2D sketch of the vibrator mount in the home-built TIRF microscope with flat-field illumination. All measurements are in millimetres. R – radius.

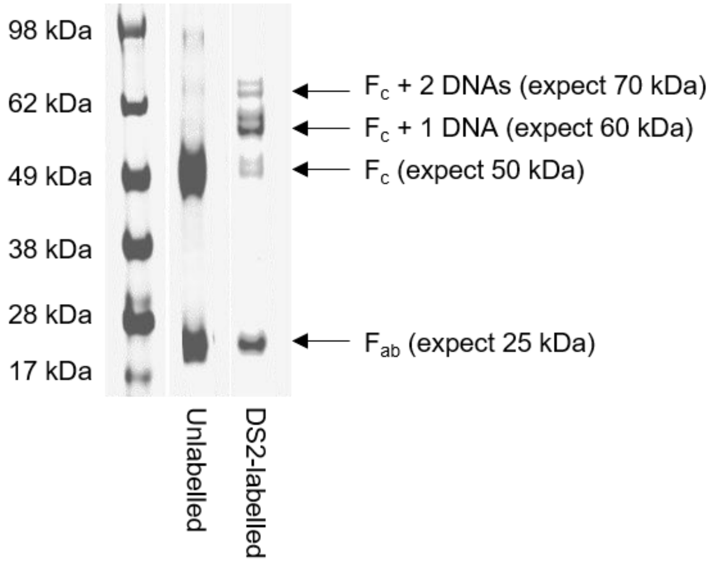

Figure S3. Characterization of DNA-labelled mouse anti-rabbit antibody by SDS-PAGE under reducing conditions.

Supplementary tables

**Method**	**Wavelength**	**Ratio of power at focal plane to power at source**
Free space launching	405 nm	7%
Free space launching	488 nm	6%
Free space launching	561 nm	12%
Free space launching	640 nm	9%
MMF	405 nm	55%
MMF	488 nm	55%
MMF	561 nm	56%
MMF	640 nm	46%

Table S1. Comparison between transmission efficiencies in a free space launching system (with the beam sufficiently expanded to simulate a flat-field) and the square-core MMF.

**Code**	**Sequence (5' - 3')**	**Application**
DBCO-DS2	DBCO TEG-TTATCTACATATTTTTTTTTTTTTTTTTTTT	Labelling of mouse anti-rabbit antibody
IS2-cy3B	TATGTAGATC-cy3B	Cellular DNA-PAINT
Aptamer-DS1	GCCTGTGGTGTTGGGGCGGGTGCGTTATACATCTA	AD-PAINT
IS1-cy3B	CTAGATGTAT-cy3B	AD-PAINT

Table S2. List of oligonucleotides and their applications. Underlined nucleotides are the complementary sequences that bind transiently to the docking strand and give a 9 bp DNA duplex during DNA-PAINT imaging. DBCO TEG – dibenzocyclooctyne tetraethylene glycol
